# Case of Dysphagia

**Published:** 1802-07

**Authors:** 

**Affiliations:** Kegworth


### *Case of Dysphagia. Communicated by* Dr. Stevenson, *of Kegworth.*

THE fuccefsful iffue of the following formidable cafe of Dyfphagia, effected by a peculiar mode of treatment, together with the annexed remarks, will not, I perfuade myfelf, be deemed irrelevant to the fpirit and defign of a publication, which has for its object the advancement of medical fcience, and confequently the alleviation of human mifery.

Mrs. Wagdin, Trent Lock, Derbyfhire, the fubject of the fubfequent communication, is forty years of age, of a thin fpare habit, and irritable temperament.

She dates the origin of her complaints from a violent attack of cynanche maligna near twelve years ago, to the contagion of which ſhe was expoſed almoſt immediately after her recovery from a ſevere parturition, During the whole term of, and indeed for ſome years previous to, utero-geſtation, her health was extremely delicate, and at times interrupted by alarming pulmonic affections, which it was feared would terminate in phthiſis. The moſt prominent features of her diſorder from that period till the expiration of more than three years, were a ſlight though progreſſively increaſed difficulty of ſwallowing, accompanied with ſome degree of ſoreneſs, and an augmentation of the ſalival excretion. With a view to the palliation of theſe ſymptoms, ſhe was directed to have occaſional recourſe to aperients, leeches, bliſters, and gargles. By this time, however, the difficulty of deglutition had become ſo alarmingly exaſperated, that ſhe was no longer capable of ſwallowing ſolids, even of the magnitude of a field pea. In this ſituation ſhe put herſelf under the direction of Dr. Smith, late of Nottingham, who very judiciouſly preſcribed mercurials. A moſt ſevere ſalivation was the conſequence, under which ſhe laboured for the protracted ſpace of three months. By this method the ſymptoms were ſo conſiderably alleviated, that ſhe was capable once more of ſwallowing ſoft and well comminuted ſolids. But though thus reſcued from her impending fate, the *remedy* was productive of effects no leſs formidable. I allude to exceſſive debility, frequent ſyncope on the leaſt motion, colliquative ſweats, her ſyſtem being greatly emaciated, and a prey to hyſterical paroxyſms. By the aid of proper dietetical management, as the complaint it was vainly hoped was ſubdued, her attendants flattered themſelves ſhe might ſtill ſurvive even this ſevere conteſt. Alas! no ſooner were her drooping ſpirits reanimated by the ſenſible acquiſition of renovated vigour, than the fond expectations ſhe had cheriſhed became depreſſed by a viſible return of her former impediment to ſwallowing.

During the laſt ſeven years, ſhe found herſelf reduced to the ſad neceſſity of ſupporting a miſerable exiſtence by means of liquid aliment, ſuch as ſoups, milk, &c. I ſaw her for the firſt time in the beginning of October, 1801, in conſequence of her having been choaked by inadvertently attempting to ſwallow a morſel of biſcuit ſoaked in tea, not equal in ſize to a grain of wheat. Several efforts indeed now became neceſſary to enable her to ſwallow a tea ſpoonful of any liquid, a part of which regurgitated ſeveral times before the whole deſcended into the ſtomach, and the pain produced by the effort was ſo ſevere, as not unfrequently to uſher in a general convulſive agitation of ſome continuance. She had then a dejected emaciated appearance, ance, a quick pulfe, and other hectic fymptoms, and was harraffed by an almoft inceffant ptyalifm, more particularly urgent during the earlier part of the day, at which time fhe was always hoarfe. The breathing was much incommoded when fhe reclined on a fofa or bed, which concurred with the other fymptoms in rendering her nights very reftlefs. Her bowels were habitually inactive. She expreffed a keen fenfe of hunger, but her cafe in this refpect was fimilar to the poetic fiction of Tantalus, alluded to by Horace,

> " Tantalus a labris fitiens fugientia captat
> " Flumina. "

Though equally alive to all the wants of fainting nature, and folicited to relieve them by a copious board, fhe was doomed to experience the extremity of *feeling*, without the poffibility of *gratifying* thofe fenfations. There was not any external tumefaction of the thyroid gland, nor could the obftructed part be obferved by infpecting the fauces.

Dyfphagia in this inveterate ftage, has I believe hitherto almoft invariably bidden defiance to the beft directed medical expedients; and the fingularity of the cafe will, I truft, be deemed a fufficient apology for the minutenefs of its defcription. Indeed feveral eminent practitioners who had, previous to myfelf, been confulted, were unanimoufly of opinion, that fhe muft fhortly and inevitably perifh from inanition. It is not my difpofition, however, to abandon hope whilft life remains; and under the fanction of that maxim of Celfus,

> " Melius eft anceps remedium quam nullum, "

I propofed to her, as a dernier refort, to have recourfe to mechanical dilatation, a practice none of the faculty had before even fuggefted. Senfible that if not fpeedily relieved, fhe muft fall a victim to this relentlefs difeafe, fhe agreed to fubmit implicitly to any plan from the adoption of which the fmalleft profpect of fuccefs might rationally be anticipated. Several confiderations determined me to refort to this method.

Firft, The fuppofition that the complaint originated in a contraction of the circular fibres of the œfophagus, conftituting a difeafe clofely analogous to urethral ftrictures.

Secondly, That in fome inftances of this malady, when incipient, that had fallen under my care, the fubjects of which were liable, from attempting to fwallow folids not fufficiently mafticated, to be occafionally choaked, the probang had been introduced not only with perfect fafety, but that by feveral repetitions of the operation, the individuals, inftead of a palliative, have eventually though unexpectedly obtained a radical cure of the ftricture.

 Thirdly,

Thirdly, Mercury, firſt recommended by the ingenious and learned Dr. Munckley, had in this dreadful example been tried, and though it very much mitigated for a time, yet proved ineffectual in eradicating the diſeaſe, notwithſtanding the remedy exerted its peculiarly pernicious influence on her irritable ſyſtem.

And fourthly, That the diſorder, if not ſpeedily alleviated, muſt prove fatal; and this circumſtance juſtified me in the application of even an ambiguous remedy.

In purſuance of theſe ideas, I firſt cautiouſly introduced a common bougie into the lower part of the pharynx. In this place, a powerful reſiſtance that occurred, and which occaſioned my inſtrument on the application of ſomewhat forcible preſſure to bend in various directions, ſeemed to confirm my theory of the nature of the diſeaſe. Thus foiled, I ventured to ſubſtitute a ſmall probang copiouſly charged with oil. It was not without ſteady and continued efforts that this operation was made to dilate the ſtricture. After having overcome this obſtacle, the inſtrument deſcended without much difficulty till it reached, I ſuppoſe, the lower portion of the œſophagus near the cardia, when a ſecond impediment announced the exiſtence of another ſtricture. The ſame meaſures however at length availed in enabling the probang alſo to force a paſſage through this contracted part, when it ſuddenly paſſed into the ſtomach. The inſtrument having been deliberately withdrawn, as ſoon as Mrs. W. had ſomewhat recovered from the irritation and fatigue produced by this proceſs, I gave her ſome gruel which ſtood ready, in order that ſhe might aſcertain, by ſipping leiſurely a ſmall quantity, whether any benefit had accrued from the operation. Upon attempting to ſwallow, ſhe found the former impediment removed, and continued drinking till ſhe had conſumed at leaſt half a pint of the liquid with the greateſt facility as to the power of deglutition, though of courſe ſome ſoreneſs muſt have exiſted.

Apprehending that the paſſage would not by the ſmall inſtrument employed, be ſufficiently dilated to admit of the ready ingurgitation of ſolids, the operation was repeated with a larger inſtrument three ſucceſſive times, a few days being ſuffered to intervene between each, in order that the topical pain might be allayed by the exhibition of oily linctuſes and aperients, and by fomentations. The *fourth* operation enabled her to ſwallow ſolids without experiencing the ſmalleſt inconvenience, a faculty ſhe ſtill continues to exerciſe in its fulleſt extent.

By adopting a regular ſyſtem of analeptics, all the hectic ſymptoms, &c. ſpeedily ſubſided, her enfeebled conſtitution ſoon rallied, and put her in the poſſeſſion of better health than ſhe ever enjoyed during the earlier part of her life.

As

As a further teſtimony of the efficacy of mechanical means in the radical cure of this tremendous diſeaſe, permit me ſhortly to add, that the only daughter of the above mentioned lady, aged twelve years, had from her earlieſt infancy, indeed from her birth, laboured under the Dyſphagia Oeſophagia. Her conſtitution partakes much of the nervous irritability of her mother. The want of ſubſtantial food (for her exiſtence had been ſupported by the ſuction of liquid aliment alone) tended obviouſly to retard the phyſical evolution of her ſyſtem. The compleat ſucceſs which had crowned my efforts in the caſe of Mrs. W. naturally created an anxious wiſh in the parents to have the ſame means reſorted to in the preſent inſtance; of the abſolute neceſſity of which my intelligent little patient was fully ſatisfied, and readily conſented to undergo the operation, from the ſanguine expectation of deriving equal benefit. It is with ſentiments of the greateſt ſatisfaction I am authoriſed to ſtate, that the expedient has proved altogether ſalutary and efficacious. The texture of her body, which previous to the operation had ever been extremely delicate, has acquired a wonderful degree of renovated vigour, and her ſpirits, formerly ſubject to great depreſſion, have obtained ſuch a healthy flow, that ſhe can now engage in juvenile amuſements with the greateſt cheerfulneſs and vivacity.

### OBSERVATIONS.

The foregoing diſeaſe ſeems decidedly a caſe of Dyſphagia Oeſophagia ſpecies, 5th of Sauvages, ariſing from a contraction of two portions of the œſophagus. Its occurrence is by no means the moſt rare phenomenon in the diſorders incident to the human race. Abſtracted from the conſideration of pain, which is ſeldom conſiderable, (except in inveterate caſes during the act of deglutition, when a general tremulous affection follows the attempt) it conſtitutes a complaint the moſt diſtreſsingly formidable to the patient, from the apprehenſion of an eventual obliteration of the paſſage to the ſtomach. As far as my obſervations have enabled me to form a compariſon, perſons of a ſlender make and irritable temperament appear eſpecially obnoxious to its attacks; hence the female ſex are more particularly ſuſceptible of this affection. Its commencement is for the moſt part obſcure and inſidious, and its progreſs far from rapid; it ſcarcely ever ariſes from any obvious aſſignable cauſe, nor can it challenge a connection with any other diſorder. Perhaps the caſe of Mrs. W. may be conſidered as affording an exception to this poſition. It is admitted that ſhe diſcovered a difficulty of ſwallowing only poſterior to her recovery from the Cynanche Maligna; but that this diſeaſe was the oc-

D 4 caſion casion of the Dysphagia is at least problematical, as its appearance at that juncture of time might have been casual; and general experience, if appealed to, will be found to contradict this inference. No age is exclusively shielded from its dreadful shafts; however, individuals who have passed the meridian, and are in the vale of life, become proportionably oftener the victims of it. Occasional exacerbations take place, more particularly during a moist atmosphere. A catarrhal indisposition never fails to exasperate its symptoms. To these two causes co-operating, may be referred its greater urgency during the autumnal months. The genuine disease disclaims any necessary combination with the struma, on which account we seldom find any concomitant enlargement of the glands of the neck or throat. The occasionally impeded deglutition, the effect of local pressure frem bronchocele, is essentially different in its symptoms and phænomena from the contracted œsophagus. A spontaneous ptyalism, the most considerable during the earlier part of the day, and after eating, is an usual attendant of this disease. This symptom probably depends, not so much upon any specific increased action of the salival glands (except indeed during deglutition, when they are probably stimulated to a more copious secretion) as upon the saliva, when secreted, becoming accumulated in the fauces; and hence the cause, probably, of the hoarseness so common in the morning before expectoration has commenced. The bowels, as it might be expected, are usually torpid. The most characteristic diagnosis of the contracted throat, added to the difficulty in swallowing, is the regurgitation of solid, and, in very desperate cases, of liquid ingesta. The ejection does not ensue instantly upon the food being swallowed, but generally a few seconds elapse before it takes place. It may be remarked, that the longer the interval before this occurs, the more malignant proportionably is the disease. Anatomical dissections of persons who have died of this complaint explain the cause of this phenomenon. In consquence of the distension occasioned by the repeated remora of alimentary substances above the strictured part, a sac or pouch is usually formed, in which the food lodges, till by its irritation a violent straining and vomiting effect its expulsion through the mouth or nose, and sometimes both. There is only one complaint that I am acquainted with that is sufficiently imitative of this under consideration, as to be liable to be mistaken for the contracted œsophagus. What I now allude to, is a spasmodic affection of the œsophagus, frequently also extending to the trachea, to which hysterical females are particularly predisposed. But the circumstance of its attack being periodical, and of only temporary duration, and of its yielding readily, for for the moſt part, to antiſpaſmodic tonics, will ſerve to diſtinguiſh it from the diſeaſe which is the ſubject of theſe remarks. As to the ſchirrous pylorus, the aliment, beſides being ſwallowed with the greateſt facility, is reduced by digeſtion to a *pultaceous mass* before it is rejected by vomiting; which two circumſtances will always afford marks of diſtinction between it and the contracted œſophagus.

I ſhall beg leave to conclude theſe narratives and obſervations with the following inferences, clearly deducible from the foregoing premiſes;

Firſt, It appears, I preſume, pretty ſatisfactory from what has been advanced, that the caſes of Mrs. and Miſs W. were inſtances of Dyſphagia Oeſophagia, occaſioned by a gradual contraction of the circular order of muſcular fibres in the œſophagus, and not by an enlargement of the lymphatic glands, with which the poſterior part of this canal is ſtudded, as Dr. Currie, of Liverpool, imagined, in a caſe of this kind which he relates that proved fatal. This ſuppoſition is, I think, admiſſible, from the conſideration of my method of cure, which has been adopted with compleat ſucceſs.

Secondly, Theſe caſes confirm the obſervations of Dr. Hunter, that ſtrictures of the œſophagus occur chiefly near the pharynx or cardia, or both; probably from a peculiar arrangement of fibres in theſe parts performing an office ſomewhat ſimilar to that of ſphincters.

Thirdly, That the operation here recommended by mechanical dilatation, when cautiouſly conducted, is perfectly ſafe, productive of inconſiderable pain, and that of ſhort duration, and apparently of laſting effect; if not, it might be repeated without inconvenience.

Fourthly, And that (what renders it ſtill more valuable) it is applicable in the moſt inveterate ſtages of this hitherto intractable diſeaſe; in *cases* where the moſt approved remedy was capable of affording *only a temporary alleviation* of the ſymptoms.

*Kegworth*, 28 *May*, 1802.

